# BKCa promotes growth and metastasis of prostate cancer through facilitating the coupling between αvβ3 integrin and FAK

**DOI:** 10.18632/oncotarget.9559

**Published:** 2016-05-23

**Authors:** Cheng Du, Zhendong Zheng, Danqi Li, Li Chen, Na Li, Xiaomin Yi, Yang Yang, Fang Guo, Wenchao Liu, Xiaodong Xie, Manjiang Xie

**Affiliations:** ^1^ Department of Oncology, General Hospital of Shenyang Military Area Command, Shenyang, P. R. China; ^2^ Department of Oncology, Xijing Hospital, Fourth Military Medical University, Xi'an, P. R. China; ^3^ Department of Aerospace Medicine, Fourth Military Medical University, Xi'an, P. R. China; ^4^ School of Traditional Chinese Materia Medica, Shenyang Pharmaceutical University, Shenyang, P. R. China; ^5^ Department of Gynaecology and Obstetrics, First Affiliated Hospital, Jilin University, Jilin, P. R. China; ^6^ Department of Urology, PLA 105 Hospital, Hefei, P. R. China

**Keywords:** BKCa, prostate cancer, metastasis, integrin αvβ3, FAK

## Abstract

BKCa is a large conductance calcium activated potassium channel promoting prostate cancer cell proliferation, although the mechanism is not fully elucidated. In addition, whether BKCa is involved in metastasis of prostate cancer remains to be explored. Here, we report that BKCa is overexpressed in prostate cancer. BKCa expression positively correlates with Ki67 index and gleason score of prostate cancer. Upregulation of BKCa promoted proliferation, migration and invasion of prostate cancer cells. On the contrary, downregulation of BKCa inhibited growth and metastasis of prostate cancer cells both in vitro and in vivo. Moreover, the ion-conducting function of BKCa contributed moderately to prostate cancer proliferation and migration, although, this was not the primary mechanism. BKCa action was mainly mediated through forming a functional complex with αvβ3 integrin. The BKCa/αvβ3 integrin complex promoted FAK phosphorylation independent of the channel activity. Overexpression of BKCa enhanced its association with αvβ3 integrin and FAK which increased FAK phosphorylation. Conversely, disrupting the complex by downregulation of BKCa reduced FAK phosphorylation. Finally, blocking of αvβ3 integrin or p-FAK activity using LM609 or Y15 markedly abrogated BKCa-enhanced cell proliferation and migration. Taken together, these results suggest that targeting BKCa/αvβ3/FAK may inaugurate innovative approaches to inhibit prostate cancer growth and metastasis.

## INTRODUCTION

Potassium channels are transmembrane proteins that regulate electrical excitability by controlling potassium flow across cell membranes. Increasing evidence suggests that potassium channels are dysregulated in a variety of malignancies involved in cancer cell proliferation, migration and invasion [1–3]. BKCa is a large conductance calcium activated potassium channel mainly expressed in excitable cells. It modulates vasomotor and nerve excitability by regulating membrane potential and calcium signaling [4, 5]. Recent studies report that BKCa is aberrantly expressed in many neoplastic cells and participate in cell growth and metastasis [1, 6]. Specifically, the BKCa coding gene (KCNMA1) is amplified in nearly 1.9% of breast cancers. This amplification correlates with high tumor grade, high proliferation index and poor tumor-specific survival [7]. Its amplification is also found in 16% of advanced prostate cancer, although the expression profile and clinical relevance are not fully elucidated [8]. In vitro studies demonstrate that BKCa promotes the proliferation of many malignant cells, including prostate cancer, breast cancer and glioma [8–11]. Other lines of evidence suggest that BKCa regulates cell migration and invasion in breast cancer and melanoma [12–14]. However, whether these in vitro findings can be observed from in vivo models remain to be explored. Furthermore, the molecular mechanisms underlying BKCa mediated cancer cell growth and metastasis are poorly understood.

Integrin αvβ3 is a heterodimeric transmembrane receptor for extracellular matrix proteins that promotes prostate cancer cell survival, proliferation, and migration [15, 16]. One of the most important downstream signaling targets stimulated by integrin αvβ3 is the focal adhesion kinase (FAK). Following integrin engagement, FAK can be recruited to sites of integrin clustering and activated through undefined mechanisms involving FAK clustering and autophosphorylation at Y397 [17, 18]. Activated FAK promotes prostate cancer cell growth and metastasis by stimulating its downstream effectors, such as Src, PI3K/Akt and ERK1/2 [18, 19]. Recently, several lines of evidence indicate that potassium channels can form functional complex with integrins and FAK to initiate intracellular signaling events in both normal and cancerous cellular contexts [20]. For example, α9β1 integrin enhances cell migration by polyamine-mediated modulation of an inward-rectifier potassium channel in Chinese hamster ovary (CHO) and mouse embryo fibroblast cells [21]. Another study reports that formation of Kv2.1/FAK complex induces FAK activation, cell polarization and motility in CHO cells [22]. In acute myeloid leukemia cells, β1 integrin, VEGFR1 and hERG potassium channel form a macromolecular signaling complex that promotes cell migration and metastasis [23]. As a transmembrane molecule, BKCa binds with FAK and regulates mechanotransduction in osteoblasts. BKCa also interact with integrins in smooth muscle and endometrial epithelial cells, regulating arteriolar dilatation and endometrial receptivity [24–27]. However, whether BKCa is associated with integrin/FAK in cancer cells remains to be determined. The functional consequence of this interaction is also unclear.

In this study, we tested the hypothesis that BKCa promotes prostate cancer cell growth and metastasis via integrin αvβ3/FAK signaling. First, we found that BKCa was overexpressed in prostate cancer cells and tissues. Expression of BKCa was positively correlated with Ki67 index and Gleason score of prostate cancer. We next observed that upregulation of BKCa promoted prostate cancer cell proliferation, migration and invasion in vitro, whereas downregulation of BKCa inhibited cell growth and metastasis both in vitro and in vivo. Finally, we revealed that BKCa enhanced prostate cancer cell proliferation and migration via forming complex with αvβ3 integrin and increasing phosphorylation of FAK (Y397). The ionic conduction and permeation independent mechanism was further tested by using BKCa channel modulators and αvβ3/FAK signaling inhibitors. This is the first report showing that BKCa promotes cancer cell proliferation and migration through a non-canonical ion-conducting independent pathway.

## RESULTS

### Overexpression of BKCa in human prostate cancer cells

**Figure 1:**
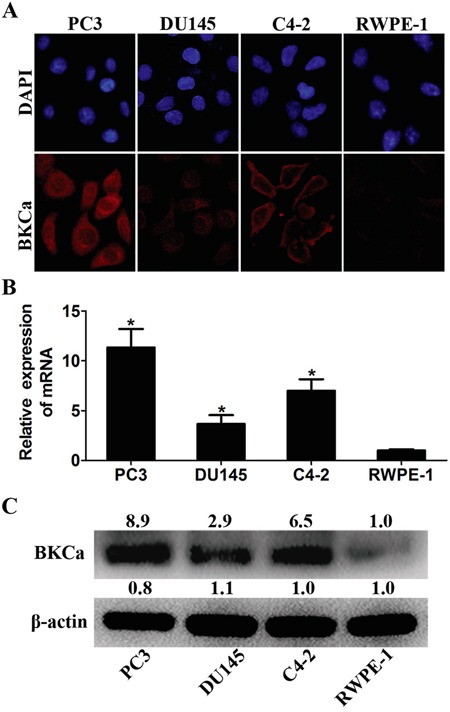
Expression analysis of BKCa in prostate cell lines **A.** Immunofluorescence staining of BKCa in cancerous prostate PC3, LNCaP and DU145 cells and normal prostate RWPE-1 cells. **B.** Real-time PCR analysis of BKCa mRNA in prostate cell lines. β-actin was used as internal control. The values were normalized to that of RWPE-1. **C.** Western blotting analysis of BKCa protein in prostate cell lines. β-actin was used as internal control and the values were normalized to that of RWPE-1. *P < 0.05.

We first examined the expression of BKCa in four prostate cell lines. Immunofluorescence assay suggested that BKCa was strongly stained in prostate cancer PC3, DU145 and C4-2 cell lines. However, it was weakly stained in normal prostate cell line RWPE-1 (Figure 1A). Increased levels of BKCa mRNA and protein in prostate cancer cell lines as opposed to the normal cell line were further documented by real-time PCR and western blotting (Figure 1B, 1C). These results are in line with previous reports showing strong expression of BKCa in cancerous PC3 and LNCaP cells as compared with non-cancerous BPH-1 cells [8]. We next examined the proliferation, migration and invasion ability of these cell lines via MTT and transwell assays. The results showed that prostate cancer cells displayed stronger cell proliferation, migration and invasion abilities than normal prostate cells (Supplementary Figure 1). This might be partly attributed to the increased expression of BKCa in cancerous PC3, DU145 and C4-2 cells than that in normal RWPE-1 cells.

### Overexpression of BKCa correlates with highly proliferative and progressive properties of prostate cancer

**Figure 2:**
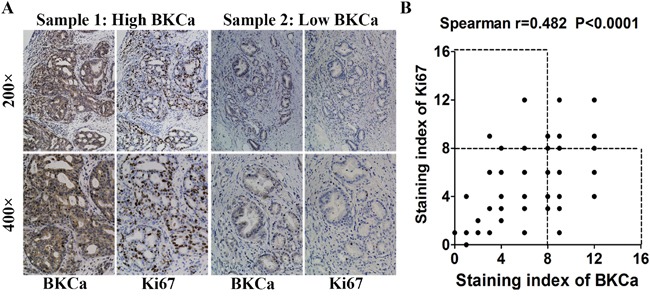
Relationship between BKCa and Ki67 expression in human prostate cancer samples **A.** Immunohistochemical (IHC) staining of BKCa and Ki67 in prostate cancer specimens. **B.** Correlation analysis of BKCa and Ki67 expression. Staining index ranges from 0 to 12. Each point represents one prostate cancer specimen.

**Figure 3:**
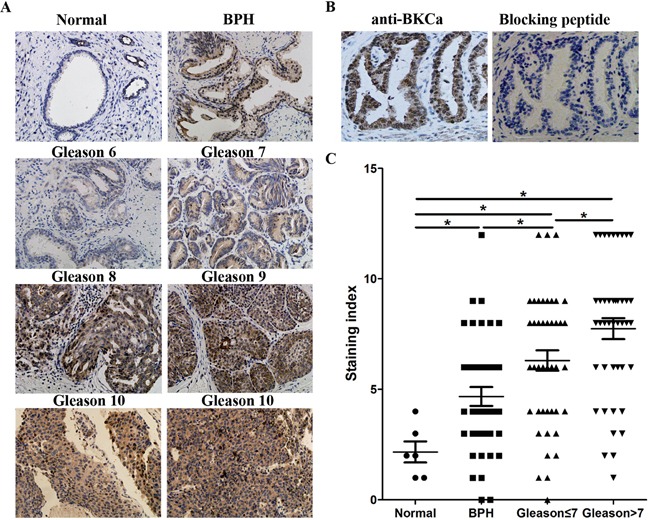
Expression of BKCa positively correlates with the Gleason score of human prostate cancer **A.** Representative images from IHC assays of archived prostate tissues. **B.** Sections were immunostained with the BKCa antibody alone or together with a BKCa blocking peptide to verify the specific staining of the antibody. **C.** Quantification of IHC staining of BKCa in normal prostate tissues, benign prostate hyperplasia tissues and prostate cancer tissues with different Gleason scores. *P<0.05, Mann-Whitney U test. *P<0.05, Mann-Whitney U test.

We collected 86 archived prostate cancer tissues and analyzed the expression of BKCa and Ki67 by immunohistochemistry. As shown in Figure 2, sections that presented high level of BKCa showed strong staining of Ki67. On the contrary, those with low BKCa expression displayed weakly staining of Ki67. Statistical analysis suggested that the expression of BKCa significantly correlated with Ki67 (Figure 2B, r=0.482, p<0.001). We further compared the expression of BKCa in normal prostate tissues (6 cases), benign prostate hyperplasia tissues (BPH, 40 cases) and prostate cancer tissues with different Gleason scores (Gleason score ≤7, 43 cases; Gleason score >7, 43 cases). As revealed in Figure 3A, BKCa was weakly or moderately stained in non-cancerous prostate tissues. However, it was highly expressed in cancerous prostate tissues. The IHC staining increased with the Gleason score of prostate cancer. The specific staining of the antibody was also verified. As shown in Figure 3B, prostate cancer section incubated with BKCa antibody combined with its blocking peptide displayed negative staining. Statistical analysis demonstrated that the expression of BKCa was significantly higher in prostate cancer tissues than that in BPH or normal prostate tissues. In addition, the expression of BKCa was more pronounced in prostate cancers of Gleason score >7 than that of Gleason score ≤7 (Figure 3C).

### Upregulation of BKCa promotes proliferation, migration and invasion of prostate cancer cells

**Figure 4:**
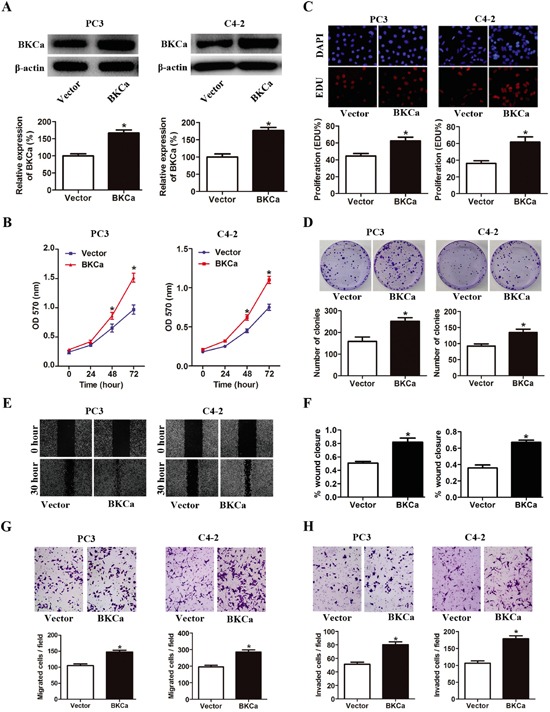
Overexpression of BKCa stimulates prostate cancer cell proliferation, migration and invasion **A.** Prostate cancer PC3 and C4-2 cells were transfected with BKCa or Vector plasmids for 48 hr. The expression of BKCa was analyzed by western blot. β-actin was used as loading control. **B.** Cell growth curves as determined by MTT assay in PC3 and C4-2 cells transfected with BKCa or Vector. **C.** Percentage of EdU positive cells determined by EdU incorporation assay (lower panel) and representative images of EdU staining (upper panel) in PC3 and C4-2 cells transfected with BKCa or Vector. **D.** Effects of upregulated BKCa on the colony-genic ability of PC3 and C4-2 cells. Representative images were shown in the upper panel and the number of colonies was illustrated in the lower panel. **E.** Overexpression of BKCa promoted prostate cancer cell migration as determined by scratch wound healing assay. The representative images of wound healing were shown in left panel. The relative wound closure was illustrated in right panel. **F-H.** Effects of upregulated BKCa on the migration and invasion of PC3 and C4-2 cells as determined by transwell assays. Representative images and mean numbers of migrated (G) and invaded (H) cells were shown in the upper and lower panel respectively. All the experiments were performed in triplicate. The data are shown as the means ± se. *P < 0.05.

Next, we investigated the effects of BKCa overexpression on cell proliferation, migration and invasion using prostate cancer PC-3 and C4-2 cell lines. As shown in Figure 4A and 4B, prostate cancer cells transfected with BKCa displayed significantly increased growth rates as compared with the control cells. The enhanced cell proliferation by BKCa upregulation was also observed in EdU incorporation and colony formation assays (Figure 4C, 4D). Moreover, BKCa-transfected prostate cancer cells demonstrated significantly increased wound healing rates by 1.6 folds as compared with cells transfected with vectors at 30 hr after scratching (Figure 4E). We also found that BKCa significantly promoted the migration and invasion of PC3 and C4-2 cells via transwell assay (Figure 4G, 4H). Taken together, these results indicated that ectopic expression of BKCa promoted prostate cancer progression in vitro by enhancing proliferation, migration and invasion.

### Downregulation of BKCa inhibits the proliferative and metastatic potential of prostate cancer cells both in vitro and in vivo

**Figure 5:**
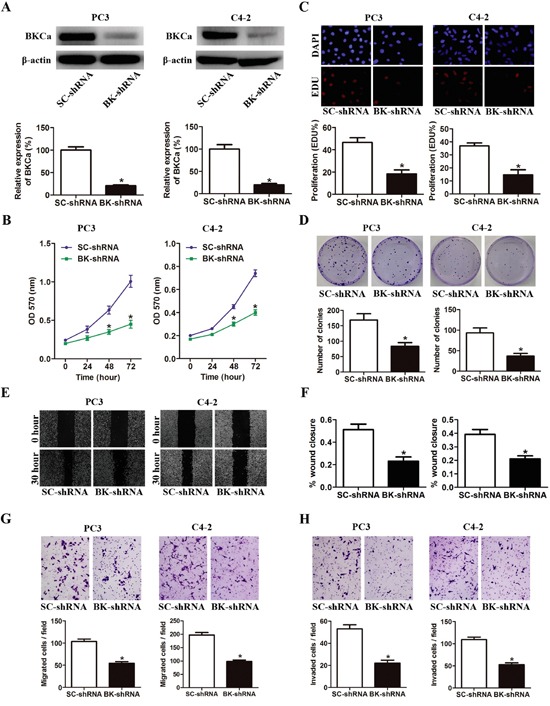
Downregulation of BKCa inhibits prostate cancer cell proliferation, migration and invasion **A.** Prostate cancer PC3 and C4-2 cells were stably infected with BKCa-shRNA (BK-shRNA) or Scrambled-shRNA (SC-shRNA). The expression of BKCa was analyzed by western blot. β-actin was used as loading control. **B.** Cell growth curves as determined by MTT assay. **C.** Percentage of EdU positive cells determined by EdU incorporation assay (lower panel) and representative images of EdU staining (upper panel) in PC3 and C4-2 cells. **D.** Effects of downregulation of BKCa on the colony-genic ability of PC3 and C4-2 cells. Representative images were shown in the upper panel and the number of colonies was illustrated in the lower panel. **E.** Reduced expression of BKCa inhibits prostate cancer cell migration as determined by scratch wound healing assay. The representative images of wound healing were shown in left panel. The relative wound closure was illustrated in right panel. **F-H.** Effects of downregulated BKCa on the migration and invasion of PC3 and C4-2 cells as determined by transwell assays. Representative images and mean numbers of migrated (G) and invaded (H) cells were shown in the upper and lower panel respectively. All the experiments were performed in triplicate. The data are shown as the means ± se. *P < 0.05.

The effects of BKCa downregulation on prostate cancer cell growth and metastasis were investigated via lentivirus mediated BKCa-knockdown system. As shown in Figure 5A, BKCa-shRNA effectively downregulated the expression of BKCa in prostate cancer cells. Cell growth was significantly suppressed by BKCa-shRNA as compared with cells infected with scrambled-shRNA (Figure 5B). Similarly, reduced expression of BKCa also significantly inhibited the DNA synthesis and colony formation ability, as indicated by declined EdU incorporation rate and reduced colony number (Figure 5C, 5D). Furthermore, BKCa-shRNA also dramatically inhibited the migration and invasion of prostate cancer cells as determined by wound healing and transwell assays (Figure 5E-5H).

**Figure 6:**
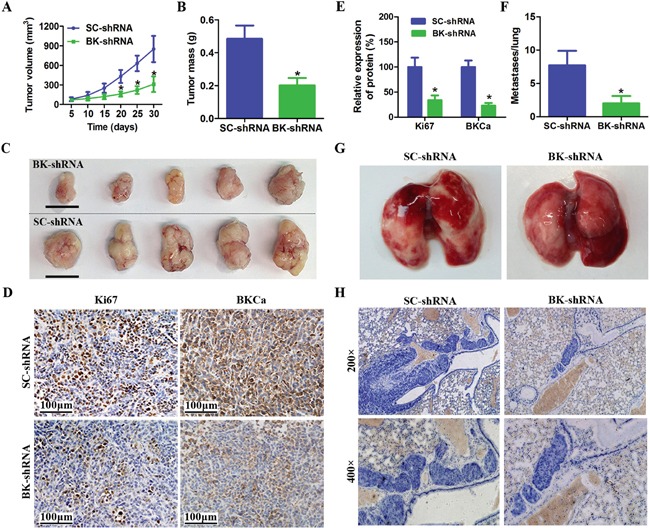
Downregulation of BKCa inhibits prostate cancer growth and metastasis in vivo **A.** Growth curves for tumor volume in mouse xenograft models. PC3 cells infected with BK-shRNA lentivirus or Scramble-shRNA were injected subcutaneously into nude mice. The tumor volume was measured every five days for one month. **B.** BKCa downregulation resulted in a decline of tumor weight. **C.** Representative images of xenografts (Scale bar 1cm). **D-E.** Immunohistochemical analysis of BKCa and Ki67 in PC3 xenografts (Scale bar 100 μm). **F.** Tumor metastasis in mouse xenograft models. PC3 cells stably infected with BK-shRNA or SC-shRNA were injected into the tail vein of nude mice. After 6weeks, the mice were killed. The metastases in the lung were calculated. **G-H.** Representative images of lungs and hematoxylin-stained sections. All of the data are shown as the means ± se. *P < 0.05.

Encouraged by these observations, we examined whether BKCa downregulation could suppress prostate cancer growth and metastasis in vivo. BKCa-shRNA or Scrambled-shRNA infected PC3 cells were subcutaneously implanted into nude mice. We found that depleted expression of BKCa significantly reduced the tumor volume and weight (Figure 6A-6C). Consistently, the IHC staining demonstrated reduced Ki67 expression in tumors with downregulated BKCa (Figure 6D, 6E and Supplementary Figure 2). We further explored the effects of BKCa downregulation on prostate cancer metastasis. PC3 cells stably transfected with BKCa-shRNA or Scrambled-shRNA were injected into the tail vein of nude mice. We observed that the number of lung metastases was significantly decreased by BKCa-shRNA compared with Scrambled-shRNA (Figure 6F-6H). Taken together, these data indicated a crucial role of BKCa for growth and metastasis of prostate cancer cells.

### BKCa promoted PC3 cell proliferation and migration mainly through an ion-conducting independent way

**Figure 7:**
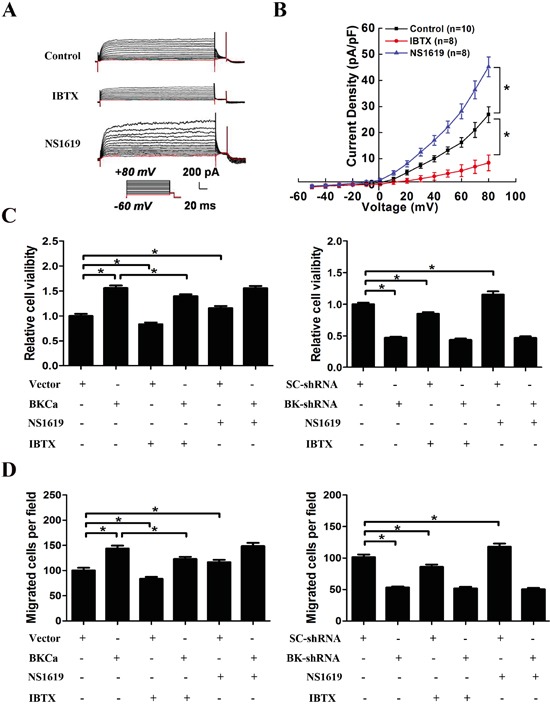
Effects of BKCa channel activator and inhibitor on the proliferation and migration of PC3 cells upon BKCa upregulation or downregulation **A.** Whole-cell K^+^ currents in PC3 cells in the presence of BKCa channel activator NS1619 (30 μM) and inhibitor IBTX (100 nM). **B.** Group data of current-voltage relationships in PC3 cells in the presence or absence of 30Mm NS1619 and 100nM IBTX. **C.** Cell viability was determined by MTT assay in PC3 cells with or without overexpression (or downregulation) of BKCa in the presence of 30μM NS1619 or 100nM IBTX for 48 hr. **D.** Cell migration was determined by transwell assay in PC3 cells with or without overexpression (or downregulation) of BKCa in the presence of 30μM NS1619 or 100nM IBTX for 24 hr. All the experiments were performed in triplicate. The data are shown as the means ± se. *P < 0.05.

As is reported that the channel activity of BKCa is associated with cancer cell proliferation and migration, we examined the response of PC3 cells to BKCa inhibitor (IBTX) and activator (NS1619). Electrophysiological analysis revealed that IBTX dramatically inhibited whole-cell current in PC3 cells, while NS1619 activated K+ currents in PC3 cells (Figure 7A, 7B). Next, we tested the effects of BKCa upregulation or downregulation on PC3 cell proliferation and migration in the presence or absence of IBTX or NS1619 by MTT and transwell assay. As shown in Figure 7C, treatment of IBTX resulted in about 16% reduction of cell growth in BKCa or vector transfected PC3 cells as compared with the vehicle treatment groups. Conversely, NS1619 increased cell proliferation by around 15% as compared with the vehicle treatment groups. Interestingly, upregulation of BKCa still increased cell growth as compared with Vector in the presence of IBTX. Similarly, when BKCa channels were blocked by IBTX, BKCa-shRNA still inhibited cell growth as compared with SC-shRNA. In line with our findings from MTT assay, the migration assays displayed similar results. Whether cells were treated with NS1619 or IBTX, up- or down-regulation of BKCa significantly promoted or inhibited cell migration. These results suggested that the ion-conducting function of BKCa contributed moderately to prostate cancer proliferation and migration, although, this was not the primary mechanism. Lots of studies have demonstrated that, in addition to their well-described function in regulating ion-permeation, potassium channels can also regulate cellular behaviors via non-canonical functions that are independent of ion-conducting function [20]. We were next prompted to explore the possible non-canonical mechanism of BKCa in promoting prostate cancer cell proliferation and migration.

### Formation of BKCa/αvβ3 integrin complex in prostate cancer PC3 cells

**Figure 8:**
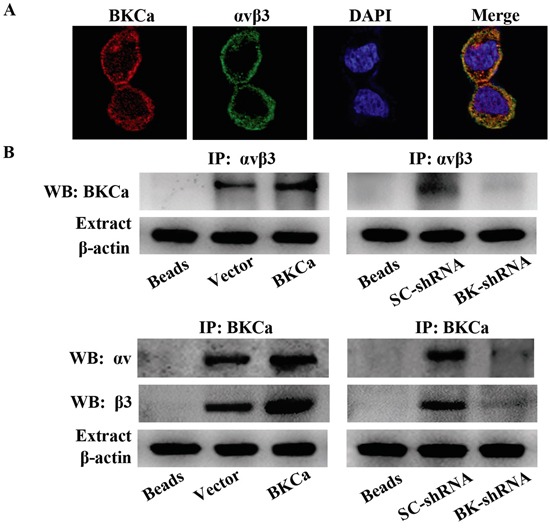
Characterization of the integrin αvβ3/BKCa complex in prostate cancer PC3 cells **A.** Immunocolocalization of BKCa and αvβ3. Representative confocal images of BKCa (red) and integrin αvβ3 (green) staining conducted in PC3 cells. Staining is more pronounced on the plasma membrane for both proteins and the merge shows that there is a co-localization (yellow to orange areas) on plasma membrane areas. **B.** Co-immunoprecipitation of integrin αvβ3 and BKCa in PC3 cells. Cells were transfected with BKCa or BK-shRNA and their corresponding negative control vectors. Proteins were extracted and immunoprecipitated using anti-BKCa or anti-αvβ3 antibodies; blots were probed with anti-αvβ3 or anti-BKCa antibodies respectively. Bead lanes contain the protein G conjugated sepharose beads used during the immunoprecipitation without the protein input. Equal amount of protein extract from each group was subjected to Western blot with β-actin as the loading control. All the experiments were performed in triplicate.

Increasing evidence shows that potassium channels and integrin can form protein complex within the plasma membrane and exert a synergic effect on intracellular signaling [28]. Thus, we determined whether BKCa channels and αvβ3 integrin are associated in PC3 cells. Immunofluorescence staining demonstrated well overlapping distributions of BKCa and αvβ3 on the membrane of PC3 cells (Figure 8A). In addition, BKCa and αvβ3 could be reciprocally immunoprecipitated by each other, indicating a physical interaction between these two molecules. We also observed that overexpression of BKCa significantly increased the interaction between BKCa and αvβ3, whereas downregulation of BKCa displayed the opposite effects. Collectively, these findings proved the formation of an integrin αvβ3/BKCa complex in PC3 cells.

As is known that among the subunits of integrin, β1, β3, and β6 are upregulated in human prostate cancer [16]. We thus examined whether β1 and β6 integrin might co-immunoprecipitate with BKCa in prostate cancer cells. The data (Supplementary Figure 3) showed that only a minor amount of BKCa was immunoprecipitated by β1 and β6 integrin in prostate cancer PC3 cells. By contrast, BKCa was obviously immunoprecipitated by αvβ3 integrin. These results suggested that the interaction between BKCa and integrin subunits is cellular context dependent. It is difficult to determine the effects of such small level of BKCa/β1 or BKCa/β6 interaction on PC3 cell proliferation and migration. Therefore, we focused our study on BKCa/αvβ3 complex.

### BKCa promotes FAK phosphorylation by recruiting it to the BKCa/αvβ3 complex

**Figure 9:**
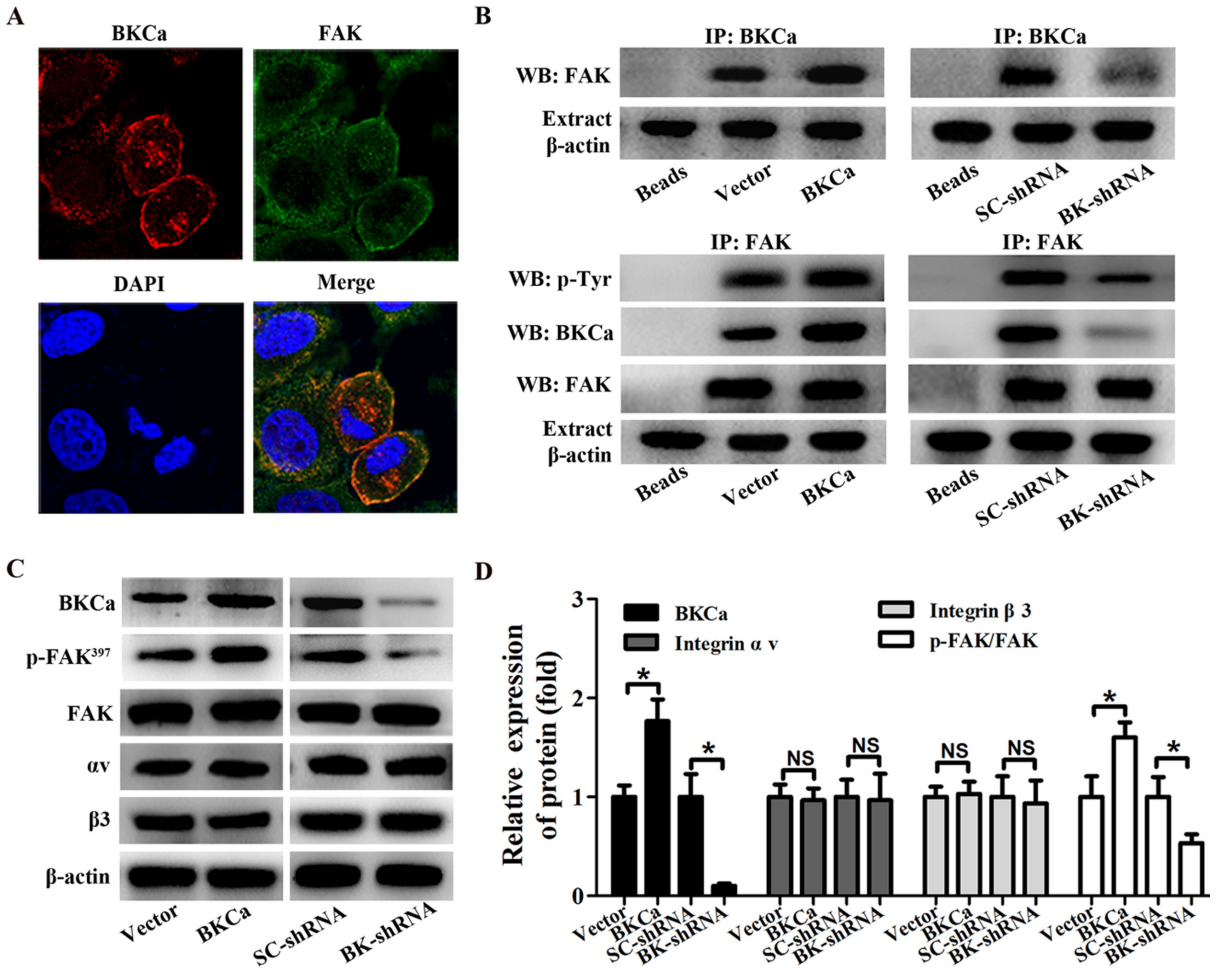
Modulation of FAK phosphorylation in PC3 cells by BKCa/αvβ3 complex **A.** Immunocolocalization of BKCa (red) and FAK (green). **B.** Co-immunoprecipitation of FAK and BKCa in PC3 cells. Cells were transfected with BKCa or BK-shRNA and their corresponding negative control vectors. Proteins were extracted and immunoprecipitated using anti-BKCa or anti-FAK antibodies; blots were probed with anti-FAK, anti-pTyr100 or anti-BKCa antibodies. Bead lanes contained the protein G conjugated sepharose beads used during the immunoprecipitation without the protein input. Equal amount of protein extract from each group was subjected to Western blot with β-actin as the loading control. **C-D.** Western blot analysis of the expression of BKCa, p-FAK (Y397), FAK and αvβ3 integrin in response to BKCa upregulation or downregulation. All the experiments were performed in triplicate. The data are shown as the means ± se. *P < 0.05.

As is well known that FAK serves as an important downstream target of the αvβ3 signaling pathway in regulating prostate cancer cell proliferation and migration. We next studied whether FAK is the downstream signaling of BKCa/αvβ3 complex. Immunofluorescence staining showed co-localization of FAK and BKCa in PC3 cells (Figure 9A). Co-immunoprecipitation assay suggested a physical interaction between FAK and BKCa. Subsequent application of an anti-tyrosine antibody revealed that the phosphorylation level of FAK was significantly increased by BKCa overexpression and decreased by its downregulation (Figure 9B). Similar results were found by western blot analysis with anti-p-FAK (397) antibody, a known auto-phosphorylation site triggered by integrin αvβ3 (Figure 9C). It was noteworthy that total levels of FAK and integrin αvβ3 were not affected by BKCa channel activity (Figure 9D). In addition, we analyzed the effects of vitronectin an αvβ3 integrin-dependent substrate, on BKCa/αvβ3/FAK integrin complex formation. The data (Supplementary Figure 4) demonstrated that PC3 cells stimulated by vitronectin showed increased BKCa/αvβ/3FAK integrin complex formation and FAK phosphorylation. However, the expression of αvβ3 integrin and FAK was unchanged. Collectively, these results revealed that BKCa might facilitate the coupling between integrin αvβ3 and FAK by recruiting FAK to the transmembrane complex of BKCa/αvβ3 integrin.

### Inhibition of integrin αvβ3/FAK signaling abrogates BKCa-enhanced proliferation and migration

**Figure 10:**
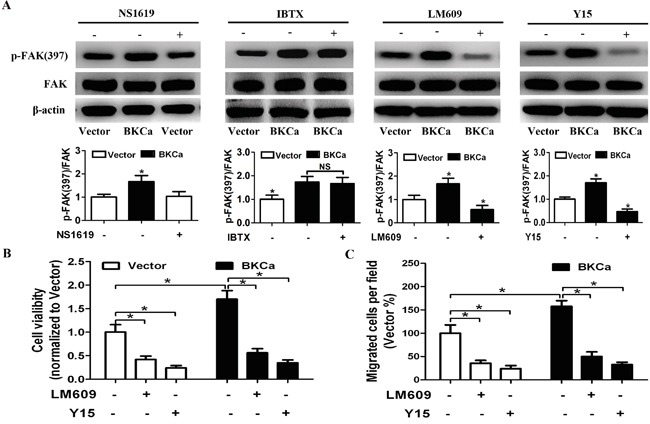
Suppression of integrin αvβ3/FAK signaling abrogates BKCa-enhanced proliferation and migration **A.** Western blot analysis of FAK and p-FAK(397) in BKCa or Vector transfected prostate cancer PC3 cells treated with NS1619 (30 μM), IBTX (100 nM), LM609 (10 μg/ml) or Y15 (10μM) for 24 hr. **B.** MTT assay showed that BKCa-enhanced cell viability was abrogated by inhibition of the αvβ3/FAK signaling. **C.** Migration assay revealed that BKCa-enhanced cell migration was abrogated by inhibition of the αvβ3/FAK signaling.

To further understand the physiologic importance of BKCa/αvβ3 complex in activating FAK signaling, we studied the effects of altering BKCa channel activity or blocking αvβ3 on downstream signaling. As shown in Figure 10A, phosphorylation of FAK was not affected by NS1619 or IBTX. On the contrary, when cells were treated with LM609 (αvβ3 integrin blocking antibody) or Y15 (FAK inhibitor), BKCa-enhanced phosphorylation of FAK was effectively abolished. Consistently, BKCa induced cell growth and migration were also abrogated by LM609 or Y15 (Figure 10B, 10C and Supplementary Figure 5). These data collectively suggested that BKCa stimulated prostate cancer cell proliferation and migration via forming complex with integrin αvβ3 and activating FAK.

## DISCUSSION

In this study, we find that BKCa is overexpressed in prostate cancer cells and tissues. BKCa upregulation promotes prostate cancer cell proliferation, migration and invasion. BKCa downregulation inhibits the above mentioned malignant activities and suppresses the in vivo growth and metastasis of prostate cancer cells. Furthermore, BKCa induces prostate cancer cell growth and metastasis possibly through forming functional complex with integrin αvβ3 and activating FAK.

Potassium channels are often abnormally expressed in various cancer cells, participating in cancer proliferation, apoptosis, migration, invasion and angiogenesis [1–3]. Among the large family of potassium channels, BKCa is also involved in promoting cancer cell proliferation, migration and invasion [4, 6]. Abdallah Mound et al. recently found that ATP induced cell proliferation could be effectively blocked in MCF-7 cells by downregulation of BKCa [10]. Another study showed that downregulation of BKCa significantly reduced invasion and transendothelial migration of breast cancer cells [13]. In melanoma, BKCa encoding gene KCNMA1 was found to be the target of miRNA-211. Ectopic expression of miR-211 resulted in significant reduced growth and invasiveness [14]. In prostate cancer, several studies also revealed that genetic suppression or pharmacological inhibition of BKCa channels impaired cell proliferation [8, 10]. In line with the above mentioned studies, we observed that downregulation of BKCa significantly inhibited the proliferative and metastatic potential of prostate cancer cells both in vitro and in vivo. Conversely, ectopic expression of BKCa enhanced the prostate cancer cell proliferation, migration and invasion of prostate cancer cells. These results were further supported by the clinical data that BKCa positively correlated with Ki67 index and gleason score of prostate cancer. Interestingly, Beatrice Cambien et al. reported that silencing of BKCa exhibited antitumoral properties in osteosarcoma in vivo. This was possibly because BKCa changed tumor microenvironment through the modulation of both chemokine expression and leukocyte infiltration [29]. These conflicting findings imply that the functional role of BKCa in cancer biology is cellular context dependent.

Potassium channels regulate cancer cell behaviors through both ion permeation–dependent and ion permeation-independent modes [20]. To unravel the molecular mechanism of BKCa induced cell growth and metastasis, we upregulated or downregulated its expression in PC3 cells in the presence of its activator NS1619 or inhibitor IBTX. To our surprise, ectopic expression of BKCa stimulated cell proliferation and migration even when the channel activity was blocked by IBTX. On the other hand, cell proliferation and migration were significantly suppressed by BKCa-shRNA, regardless of the treatment of IBTX or NS1619. Therefore, it seemed unlikely that the actions of BKCa on proliferation and migration of prostate cancer cells could be attributed simply to changes in membrane potential. BKCa possibly exerted oncogenic activities through a novel ion-conducting independent mechanism.

Potassium channels can interact with membrane protein or intracellular protein to initiate signaling cascades [20]. The best-illustrated example is the forming of a macromolecular complex by HERG channel, VEGFR-1 and β1 integrin in leukemia. The crosstalk among these proteins activates both MAPK and PI3K/AKT signaling pathways and confers a pro-migratory phenotype of leukemia both in vitro and in vivo [23]. Another study reported that transfection of non-conducting EAG channel into NIH 3T3 fibroblasts or C2C12 myoblasts significantly increased the MAP kinase activity and cell proliferation [30]. BKCa is also functionally coupled with other signaling molecules to exert concerted functions. In cancer cells, BKCa can band with Cav3.2, caveolin-1, IP3R3 and FAK to regulate cancer biological behaviors [9, 10, 12, 24]. In non-neoplastic cells, BKCa also interplay with many membrane proteins or signaling molecules, including Syk, c-Src and α5β1 integrin, to regulate physiological or pathological activity in different cellular context [25, 26, 31]. Given the reported interactions between BKCa and multiple signaling proteins, we also investigated its potential interaction with these molecules in prostate cancer. First, we found BKCa was physically associated with integrin αvβ3 and FAK in PC3 cells. Their interaction could be enhanced (or disrupted) by BKCa upregulation (or downregulation). Importantly, elevated BKCa led to increased phosphorylation of FAK, while downregulation of BKCa exerted the opposite effects. However, the expression of FAK or integrin αvβ3 was not changed. In addition, BKCa-stimulated FAK activation was not affected by its specific pharmacological modulators, indicating a non-canonical ion-conducting independent role of BKCa. Finally, we found that BKCa-induced FAK phosphorylation was mainly responsible for increased cell proliferation and migration. These effects were markedly abrogated in the presence of αvβ3 blocking antibody or FAK inhibitor.

**Figure 11:**
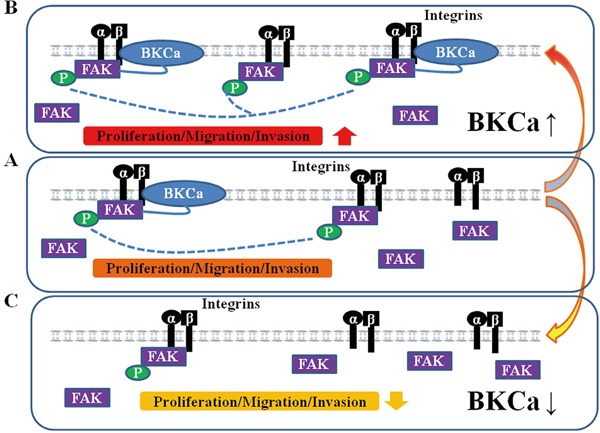
Proposed mechanism for the role of BKCa in regulating prostate cancer cell proliferation, migration and invasion **A.** Under normal conditions, BKCa forms complex with integrin αvβ3 and triggers FAK activation/phosphorylation. The activated FAK promotes prostate cancer cell proliferation, migration and invasion. **B.** Overexpression of BKCa enhance its interaction with integrin αvβ3, recruits more FAK to the integrin αvβ3/BKCa complex and promotes activation/phosphorylation of FAK, resulting in increased cell proliferation, migration and invasion. **C.** Downregulation of BKCa disrupts the integrin αvβ3/BKCa complex and abolishes BKCa-mediated αvβ3/FAK coupling. Thus, BKCa-induced cancer cell proliferation, migration and invasion are counteracted.

In summary, our results identify a hitherto unknown mechanism of BKCa-mediated prostate cancer cell growth and metastasis, involving integrin αvβ3/FAK signaling pathway (Figure 11). Thus, BKCa and integrin αvβ3/FAK signaling may serve as therapeutic targets for prostate cancer harboring elevated BKCa.

## MATERIALS AND METHODS

### Cell culture

Human prostate PC3, DU145 and RWPE-1 cell lines were purchased from ATCC. Human prostate cancer cell line C4-2 was obtained from Dr. Xiaomin Yi (Fourth military medical University, Xi'an, China). Cells were cultured in DMEM-F12 (for PC3 and DU145) or RPMI 1640 medium (for C4-2 and RWPE-1) supplemented with 10% fetal bovine serum (Gibco, NY, USA), 100μg/ml streptomycin and 100 U/ml penicillin at 37°C in a humidified incubator with 5% CO_2_.

### RNA isolation and real-time PCR

Total RNA was isolated using RNAiso Plus (Takara, Dalian, China) and reversely transcribed with a reverse transcription polymerase chain reaction (PCR) kit (Takara, Dalian, China) as described by the manufacturers. Real-time PCR was done using specific primers (BKCa: 5′-TGGCAGAGTCCTGGTTGTC-3” and 5′-CGCAAGCC GAAGTAGAGAAG-3′; β-actin: 5′-GGAGATTACTGCCCTGGCTCCTA-3′ and 5′-GACTCATCGTACTCCTGCTTGCT-3′) with the QuantiTect SYBR Green PCR Kit (Takara, Dalian, China) as previously described [32].

### Plasmids transfection and retroviral infection

The DNA plasmid pIRES-BKCa was a gift from Dr. J. D. Lippiat (Dept. of Cell Physiology and Pharmacology, University of Leicester, Leicester, UK) [12]. The vector plasmid was used as negative control. Transfection was performed using Lipofectamine 2000 and Opti-MEM Reduced-Serum medium (Invitrogen, Carlsbad, CA, USA) as described previously. Cells were transfected with plasmid for 48 h before functional assays were carried out. BKCa downregulation was achieved using the retroviral expression vector psiLv-U6 (GeneCopoeia, Rockville, MD, USA). The shRNA sequence against BKCa was 5′-GTGGGTCTGTCCTTCCCTACT-3′. Viruses were generated and used to infect target cells. Stable clones were selected using puromycin.

### Western blot

Total proteins were extracted with the M-PER mammalian protein extraction reagent with protease and phosphatase inhibitors (Thermo, Rockford, IL, USA). The protein concentration of the supernatant was determined by BCA assay kit (Thermo). The following antibodies were used: rabbit anti-FAK, rabbit anti-integrin αv and β3, mouse anti-β-actin, rabbit anti-pTyr100, peroxidase-conjugated goat anti-rabbit or anti-mouse IgG (all from Cell Signaling Technology, MA, USA) and rabbit anti-BKCa (Alomone Labs, Jerusalem, Israel).

### Immunohistochemistry

This study was approved by the Ethics Committee of General Hospital of Shenyang Military Area Command with informed consent from the patients. Immunohistochemical analysis was performed as described previously [32]. Sections were incubated with anti-BKCa (1:200, Alomone Labs) or anti-Ki67 (1:400, Cell Signaling Technology). Immunostaining was assessed by combining the scores of the proportion of positively stained cells (0, no positive tumor cells; 1, 1%-10% positive tumor cells; 2, 11%-25% positive tumor cells; 3, 26%-50% positive tumor cells; 4, 51%-100% positive tumor cells) and the intensity of staining (0, no staining; 1, week staining; 2 moderate staining; 3 strong staining).

### Immunofluorescence staining

Immunofluorescence staining was performed as described previously [12, 32]. The following antibodies were used: rabbit anti-BKCa and mouse anti-αvβ3 (Millipore, Billerica, MA, USA); mouse anti-BKCa and rabbit anti-FAK (Abcam, Cambridge, MA, USA). Images were taken with confocal microscope.

### Cell viability assay

Cell viability was measured by MTT (methylthiazolyltetrazolium) assay as described previously [12]. Cells were treated with 100 nM IBTX (BKCa channel inhibitor, Sigma, St. Louis, MO, USA), 30μM NS1619 (BKCa channel activator, Sigma), 10 μg/ml LM609 (αvβ3 integrin blocking antibody, Millipore) or 10μM Y15 (a FAK inhibitor, Abcam) for 72 hr.

### EdU incorporation assay

EdU incorporation assay was performed as described previously using Cell-Light™ EdU Apollo^®^488 In Vitro Imaging Kit (RBbio, Guangzhou, China) [32]. EdU-labeled cells were counted in ten randomly selected fields under a fluorescent microscope.

### Colony formation assay

Cells were seeded into a 60mm cell culture dishes (300 cells/dish) and cultured in 10 ml of complete medium containing 10% FBS for two weeks. Afterwards, cells were fixed with 4% paraformaldehyde and stained with 1% crystal violet. The colonies were counted.

### Scratch wound assay

Cells were seeded into 6-well plates at a density of 2×10^6^/well and grown overnight to ensure confluence. Cell monolayers were then scratched using a sterile 200 μl micropipette tip and washed with fresh medium to remove the debris. Images were taken with an inverted microscope at 0 and 30 hr after the scratch. Gap distance between the two margins of wound was measured with ImageJ software. Wound closure was calculated as follows: (gap distance at 0 hours − gap distance at 30 hours) / gap distance at 0 hours.

### Migration and invasion assays

Migration and invasion assay was carried out as described previously with Matrigel uncoated (for migration) and coated (for invasion) transwell system (BD, NJ, USA) [12]. Both assays were quantified as the number of cells counted in 10 random fields at 200×magnification. Cell migration was also examined under the treatment of the above-described reagents for 24 hr (100 nM IBTX, 30μM NS1619, 10 μg/ml LM609 or 10μM Y15).

### Nude mice study

Nude mice study was carried out as described previously using PC3 cells transfected with BKCa-shRNA or Scrambled-shRNA [32]. The expression of Ki-67 and BKCa was examined with immunohistochemistry. For the in vivo lung metastasis assay, cells were injected into the lateral tail veins of nude mice. The number of nodules was counted and the paraffin-embedded sections of lung tissues were subjected to H&E staining. All animal studies were performed according to National Guidelines for the Care and Use of Laboratory Animals with the approval of the Animal Ethics Committee of the General Hospital of Shenyang Military Area Command.

### Immunoprecipitation

Immunoprecipitation was performed using Pierce classic IP kit (Thermo, IL, USA) according to the manufacture's instructions. The immune complexes were subjected to western blot as above-described using the indicated primary antibodies. Cell extract incubated with equal amount of PBS was used as negative control, and submitted directly to immunoprecipitation and western blot. Equal amount of protein extraction was subjected to western blot with β-actin as the internal control.

### Patch clamp experiments

Whole-cell patch-clamp recordings were performed using an amplifier (CEZ-2300, Nihon Kohden Co., Tokyo, Japan) as previously described [12]. Patch clamp software (Axon Instruments, CA, USA) was used to control voltage, as well as to acquire and analyze data.

### Statistical analysis

Statistical analysis was carried out using the SPSS 13.0 statistical software package (SPSS Inc., Chicago, IL, USA). Numerical data were presented as mean ± SEM and compared with a two-tailed Student's *t* test. The IHC score of BKCa in different groups were compared with Mann-Whitney U test. The association between BKCa and Ki67 was analyzed using the Spearman correlation test. P < 0.05 was considered statistically significant.

## SUPPLEMENTARY FIGURES


