# Supplementary material for: BKCa promotes growth and metastasis of prostate cancer through facilitating the coupling between αvβ3 integrin and FAK
Source: Oncotarget. 2016 May 23;7(26):40174–88. doi: 10.18632/oncotarget.9559 (PMC5130001; doi:10.18632/oncotarget.9559)
Supplement: Supplementary file 1 [file oncotarget-07-40174-s001.pdf]

BKCa promotes growth and metastasis of prostate cancer through facilitating the coupling between αvβ3 integrin and FAK

SUPPLEMENTARY FIGURES

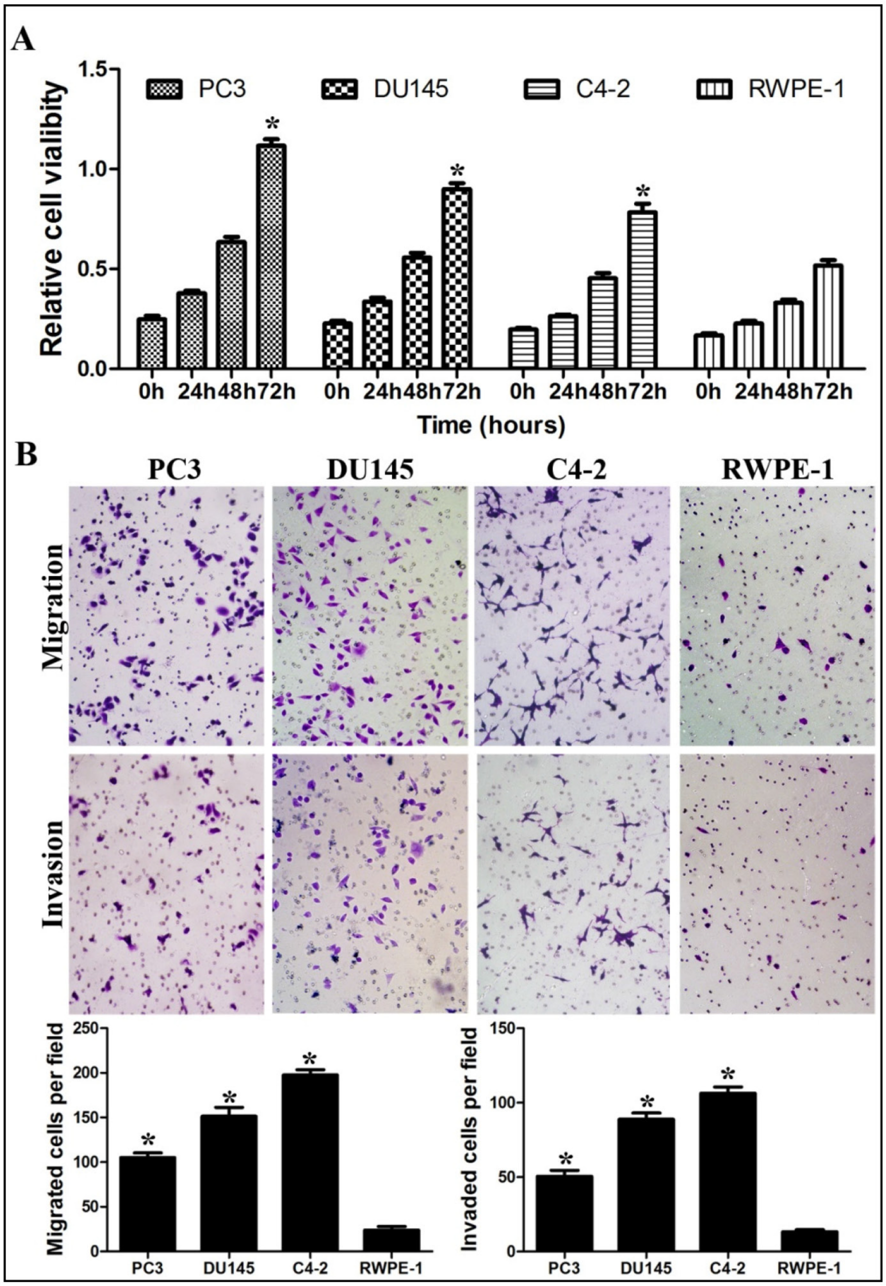

Supplementary Figure S1: Proliferation, migration and invasion ability of prostate cell lines. A. Cell proliferation was determined by MTT assay in cancerous prostate PC3, DU145, C4-2 cells and normal prostate RWPE-1 cells. **B.** Cell migration and invasion ability were determined by transwell assays in the indicated cell lines. Representative images and mean numbers of migrated and invaded cells were shown. All the experiments were performed in triplicate. The data are shown as the means ± se. * $p<0.05$ compared with RWPE-1.

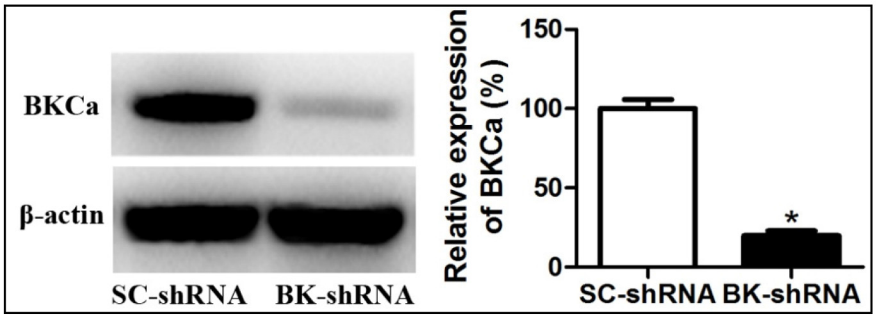

Supplementary Figure S2: Downregulation of BKCa in PC3 xenografts. Western blot analysis of BKCa expression in SC-shRNA and BK-shRNA infected PC3 cell xenografts. The experiments were performed in triplicate. The data are shown as the means ± se. *P < 0.05.

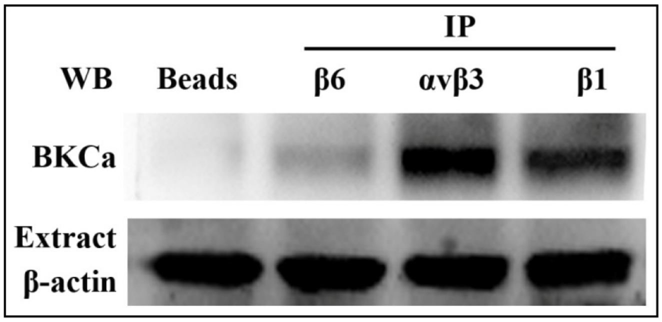

Supplementary Figure S3: Co-immunoprecipitation of integrin subunits and BKCa in PC3 cells. Proteins were extracted, immunoprecipitated and revealed using antibodies against β6, αvβ3 and β1 integrin. Bead lanes contain the protein G conjugated sepharose beads used during the immunoprecipitation without the protein input. Equal amount of protein extract from each group was loaded in Western blot with β-actin as the loading control.

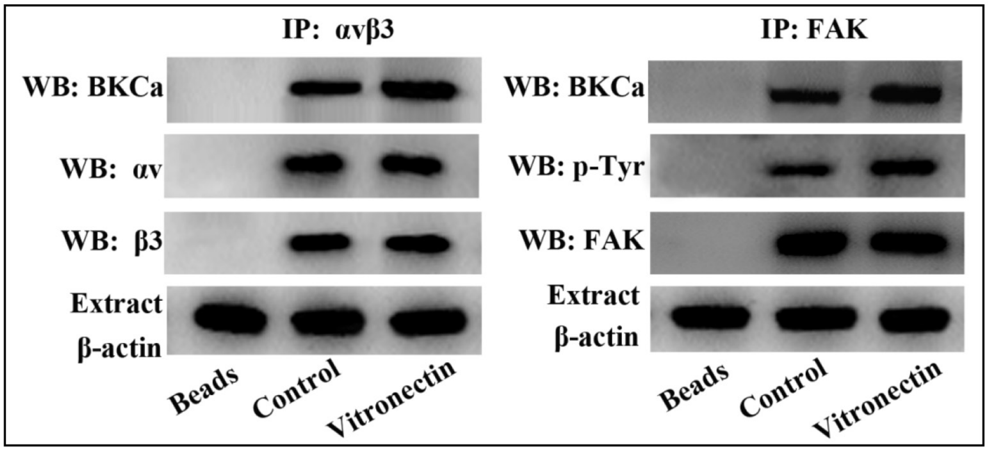

Supplementary Figure S4: Vitronectin stimulated BKCa/αvβ3 integrin interaction and FAK phosphorylation in PC3 cells. The immunocomplex was pulled down using αvβ3 integrin or FAK antibody. Cells stimulated by vitronectin (seeded on vitronection-coated dishes for 60 min) showed increased BKCa/αvβ3 integrin complex (left panel) and BKCa/FAK complex formation (right panel) and FAK phosphorylation. The expression of αvβ3 integrin and FAK proteins was unchanged. Bead lanes contain the protein G conjugated sepharose beads used during the immunoprecipitation without the protein input. Equal amount of protein extract from each group was loaded in Western blot with β-actin as the loading control.

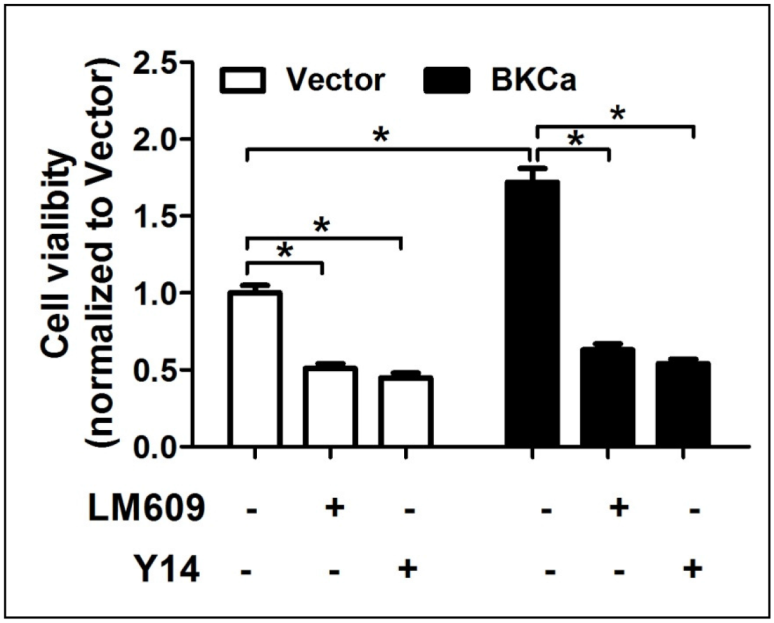

Supplementary Figure S5: Effects of BKCa channel modulators on the proliferation of PC3 cells upon BKCa upregulation or downregulation. Cell viability was determined by CCK-8 assay in PC3 cells with or without overexpression (or downregulation) of BKCa in the presence of 30μM NS1619 or 100nM IBTX for 48 hr. The experiments were performed in triplicate. The data are shown as the means ± se. *P < 0.05.
